# Effects of *PmaIAA27* and *PmaARF15* genes on drought stress tolerance in *pinus massoniana*

**DOI:** 10.1186/s12870-023-04498-z

**Published:** 2023-10-09

**Authors:** Liangliang Li, Yan Li, Wenxuan Quan, Guijie Ding

**Affiliations:** 1 Forest Resources and Environment Research Center, Key Laboratory of Forest Cultivation in Plateau Mountain of Guizhou Province, College of Forestry, Guizhou University, Guiyang, 550001 China; 2 Institute of Mountain Resources of Guizhou Province, Guiyang, 550001 China

**Keywords:** Aux/IAA, ARF, Pinus massoniana, Hormones, Drought

## Abstract

**Background:**

Auxin plays an important role in plant resistance to abiotic stress. The modulation of gene expression by Auxin response factors (ARFs) and the inhibition of auxin/indole-3-acetic acid (Aux/IAA) proteins play crucial regulatory roles in plant auxin signal transduction. However, whether the stress resistance of Masson pine (*Pinus massoniana*), as a representative pioneer species, is related to Aux/IAA and ARF genes has not been thoroughly studied and explored.

**Results:**

The present study provides preliminary evidence for the regulatory role of the *PmaIAA27* gene in abiotic stress response in Masson pine. We investigated the effects of drought and hormone treatments on Masson pine by examining the expression patterns of *PmaIAA27* and *PmaARF15* genes. Subsequently, we conducted gene cloning, functional testing using transgenic tobacco, and explored gene interactions. Exogenous auxin irrigation significantly downregulated the expression of *PmaIAA27* while upregulating *PmaARF15* in Masson pine seedlings. Moreover, transgenic tobacco with the *PmaIAA27* gene exhibited a significant decrease in auxin content compared to control plants, accompanied by an increase in proline content - a known indicator of plant drought resistance. These findings suggest that overexpression of the *PmaIAA27* gene may enhance drought resistance in Masson pine. To further investigate the interaction between *PmaIAA27* and *PmaARF15* genes, we performed bioinformatics analysis and yeast two-hybrid experiments which revealed interactions between PB1 structural region of *PmaARF15* and *PmaIAA27*.

**Conclusion:**

The present study provides new insights into the regulatory functions of Aux/IAA and ARF genes in *Masson pine*. Overexpression of *PmaIAA* gene may have negative effects on the growth of *Masson pine*, but may improve the drought resistance. Therefore, this study has great application prospects.

**Supplementary Information:**

The online version contains supplementary material available at 10.1186/s12870-023-04498-z.

## Background

The plant hormone auxin plays a pivotal role in various essential physiological processes, including cell division, differentiation, and elongation. Auxin-associated genes exert crucial regulatory functions in the growth and development of plant cells, rendering them significant targets for biotechnological approaches aimed at enhancing crop yield [1]. Previous studies have demonstrated the pivotal role of auxin in enhancing plant drought resistance, as it has been observed that drought stress triggers the activation of genes involved in auxin synthesis [2]. Auxin promotes plant root branching and potentially improves the drought resistance of plants [3]. The auxin signaling pathway includes signal recognition, expression of downstream growth hormone-related genes, and the ultimate physiological response exhibited by the plant [4]. ARF and Aux/IAAs are two of the most important transcription factors that modulate auxin responses in plants [5].

The auxin signaling pathway is highly conserved across plant species. The early auxin signaling cascade involves three protein families: the TIR1/AFB auxin co-receptors, the Aux/IAA transcriptional inhibitors, and the ARF transcription factors. In conditions of low auxin levels, IAA binds to target promoters of ARF and represses downstream gene transcription. Conversely, when auxin levels are elevated, the interaction between auxin and TIR1 leads to IAA protein degradation and activation of signaling pathways [6]. ARFs are transcription factors that modulate the expression of auxin-responsive genes. Most ARFs contain three structural domains, the amino-terminal conserved DNA structural domain DNA-binding domain (DBD), the middle region (MR) structural domain, and the carboxy-terminal Phox and Bem1 (PB1) [7]. Most Aux/IAA proteins comprise 4 structural domains: I, II, III, and IV [8, 9]. Structural domain I consist of a conserved leucine sequence. The structural domain II sequence is highly conserved and binds to the auxin receptor during signal transduction to cause ubiquitinated degradation of Aux/IAA factors, thereby modulating the expression of downstream genes [10, 11]. Structural domains III and IV form a dimer with the ARF protein PB1, thereby inhibiting the expression of auxin-responsive genes [12].

To explore the molecular mechanisms of auxin signal transduction, Aux/IAA and ARF genes have been identified and characterized in several plant species, including *Arabidopsis thaliana* [13], *Malus* [14], *Zea mays* [15], *Coix lacryma-jobi* [16], tomato [17], soybean [18], *Vitis vinifera* [19], *Brassica rapa* [20], *Triticum aestivum* [21], *sorghum* [22], *Citrus sinensis* [23] and *Cucumis sativus* [24]. Previous studies have found that the functions of Aux/IAA and ARF genes are relatively different in different plants [25]. The Masson pine, as the predominant afforestation tree in southern China, plays a pivotal role not only in forest resource development but also as a pioneering anti-stress plant species, thereby contributing significantly to ecological construction within the country [26].

The transcriptome of Masson pine under different drought treatments was determined by the previous team members and deposited in the Gen Bank Transcriptome Shotgun Assembly (TSA) database (GFHB00000000). Mining analysis of transcription factors revealed significant up-regulation or down-regulation in plant hormone signaling pathways for ARF (*c83733-g1*) and IAA (*c77087-g1*) [27]. Considering the association between Aux/IAA and ARF family genes of Masson pine with drought resistance to some extent, further comprehensive investigations were conducted on *c77087-g1* (*PmaIAA27*) and *c83733-g1* (*PmaARF15*) genes of Masson pine, encompassing gene expression, protein domain, bioinformatics, and protein-protein interaction analyses under drought and auxin treatments. This study aims to enhance our understanding of the response and interaction mechanisms of certain genes within Aux/IAA and ARF family members in Masson pine towards drought stress and exogenous hormones. These detailed analyses provide a foundation for elucidating the roles played by Aux/IAA and ARF genes in Masson pine.

## Results

### Effects of drought (D) and drought hormone (DH) treatments on gene expression

**Fig. 1:**
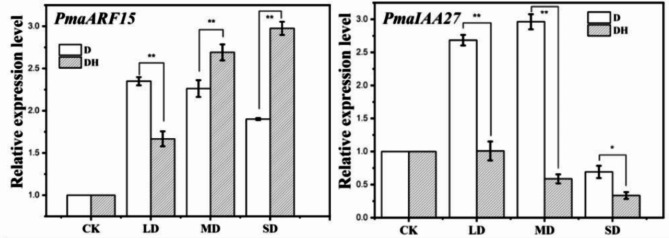
Effects of drought (D) and drought hormones (DH) on gene expression. * indicates that the difference between different temperatures at the same drought level is significant at 0.05 level;** represents a significant difference at 0.01 level; D and DH represent drought treatment and drought hormonal (IAA) treatment; CK, LD, MD and SD represent 75-80% maximum field capacity (FC)(control, CK), 55-60% FC (light drought, LD), 40-45% FC(moderate drought, MD), and 30-35% FC(severe drought, SD)

As shown in Fig. 1, the *PmaARF15* gene expression increased first and then decreased with the continuous increase of drought under drought treatment (D), reaching the maximum value in mild drought. During drought hormonal treatment (DH), the expression of the *PmaARF15* gene gradually increased with the increase of drought stress. Compared with drought treatment (D), drought hormone treatment (DH) significantly increased the expression of the *PmaARF15* gene under MD and SD, and both reached an extremely significant level. Under drought treatment (D), the expression of the *PamIAA27* gene increased first and then decreased with increasing drought stress, and reached the maximum under MD stress. Under the treatment of drought hormone treatment (DH), the expression of the *PmaARF15* gene gradually decreased with drought stress.

It can be clearly seen that under LD, MD and SD, compared with drought treatment (D), drought hormone treatment (DH) significantly reduced the *PmaIAA27* gene expression, and all reached significant difference levels, in which LD and MD reached extremely significant levels.

### Bioinformatics analysis of *PmaIAA27* and *PmaARF15*

**Fig. 2:**
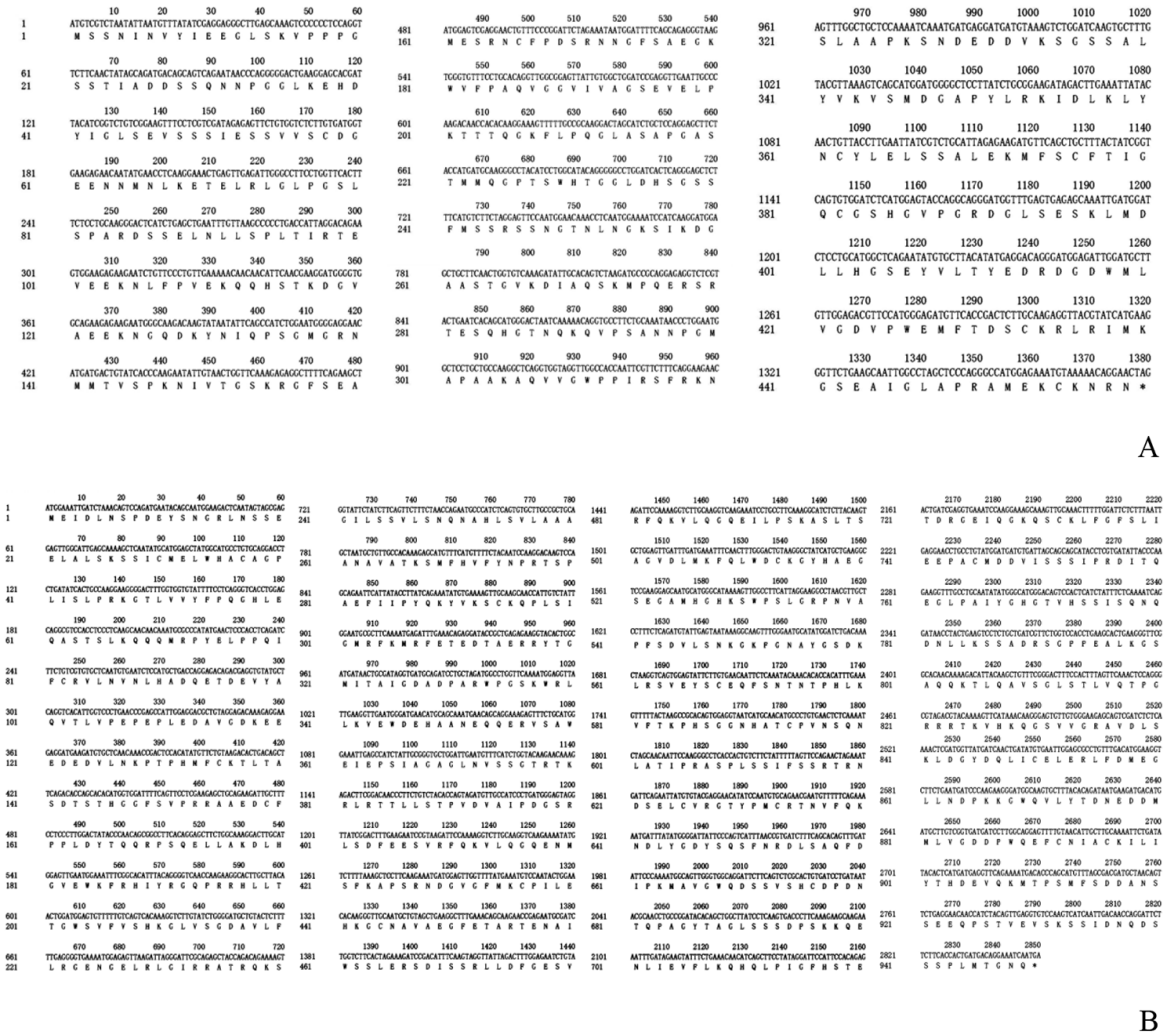
Coding sequences and derived amino acid sequences of *PmaIAA27* (**A**) and *PmaARF15* (**B**)

Open reading frame analysis: The full sequence length of *PmaIAA27* is 3055 bp, the 5’ end of cDNA contains a noncoding region (UTR) sequence length of 802 bp, and the 3’ end contains a noncoding region (UTR) sequence length of 873 bp, among which the open reading frame sequence length is 1380 bp. The 459 amino acids-encoded open reading frame is shown in Fig. 2A. The full sequence length of *PmaARF15* is 4103 bp, the 5’ end of cDNA contains the noncoding region (UTR) sequence length is 845 bp, and the 3’ end contains the noncoding region (UTR) sequence length is 408 bp, among which the open reading frame sequence length is 2850 bp. The open reading frame encoding 949 amino acids is displayed in Fig. 2B.

**Fig. 3:**
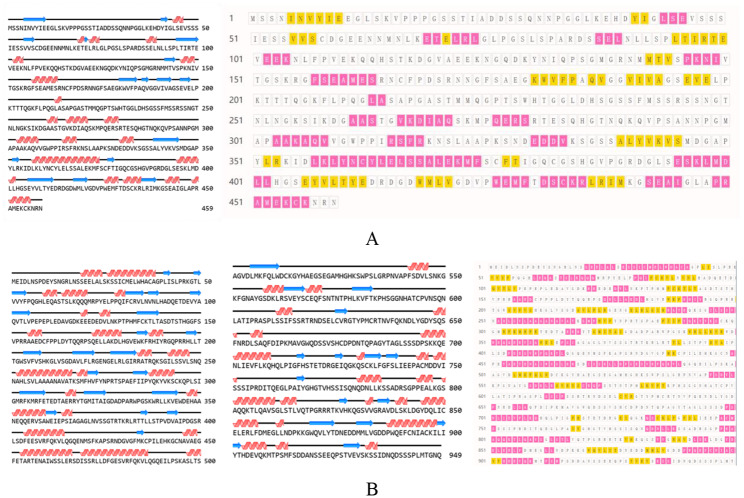
Analysis of the secondary structure of the proteins encoded by *PmaIAA27* (**A**) and *PmaARF15* (**B**). Red represents α-helix, yellow and blue arrows represents β-sheet

**Fig. 4:**
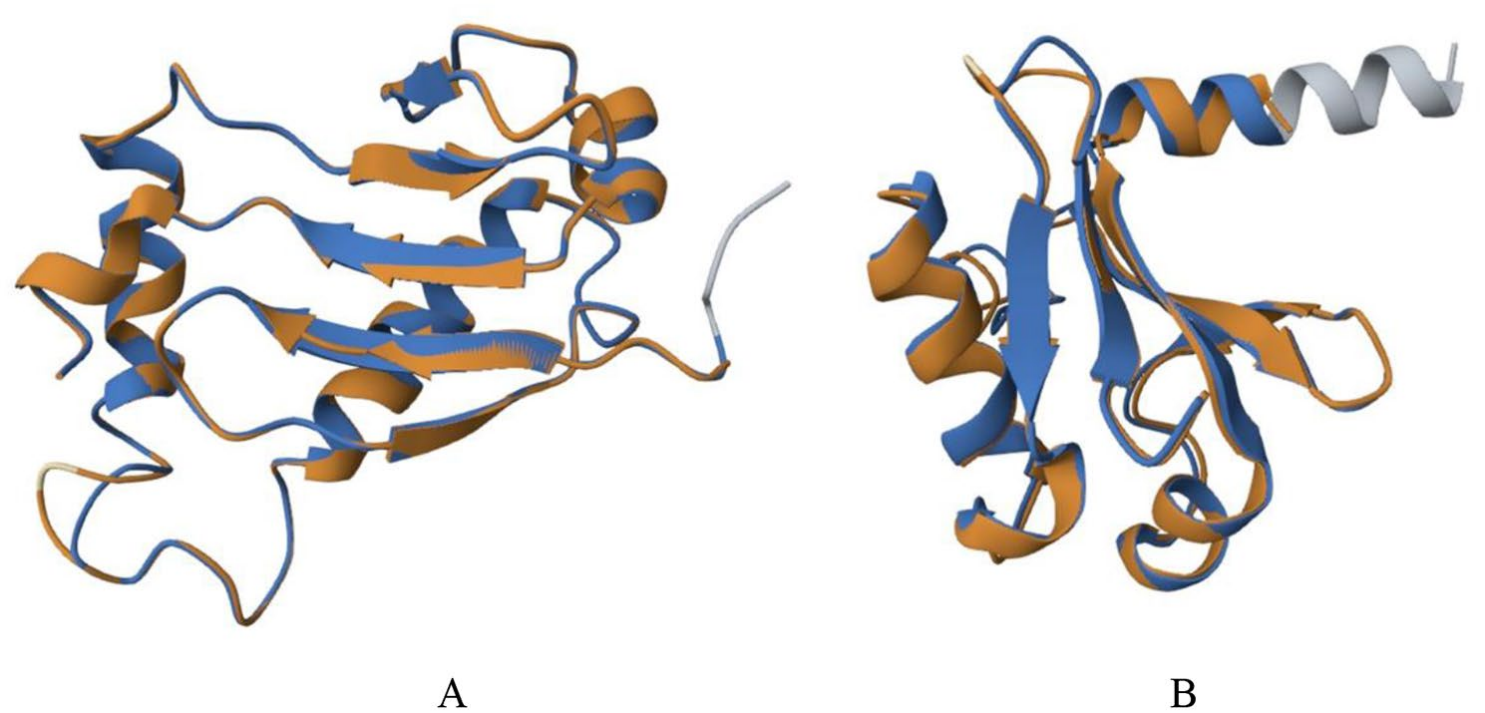
*PmaIAA27* (**A**) and *PmaARF15* (**B**) encoding protein tertiary structure homology modeling. Orange and blue are aligned amino acid residues for the target and template proteins, respectively, and all other colors are unaligned amino acid residues

Protein level 1 structure prediction: *PmaIAA27* contains 459 amino acids, the highest of which is serine (60; 13.1%), containing 50 positively charged amino acid residues and 55 negatively charged amino acid residues. The isoelectric point is 5.92, and it is an acidic protein (negatively charged) with the molecular structure formula of C_2121_H_3385_N_609_O_702_S_25_, the molecular weight of 49450.37 U, and an unstable protein. No potential transmembrane region was found in TMHMM’s prediction of the sequence. This protein has phosphorylation sites, including 47 serines (Ser), 10 threonines (Thr), and 5 tyrosines (Tyr), all of which are potential phosphorylation sites for protein kinases. The protein was predicted to be a nonsecretory protein by signALP-4.1 (Additional file 1: Fig. 4). WoLF PSORT was used to predict that this protein was most likely to exist in the nucleus (Additional file 1: Fig. 3). Secondary structure of protein of NovoPro showed that the protein had 22 α-helices and 15 β-folds (Fig. 3A). 1 H, 13 C, and 15 N Chemical Shift Assignments for Aux/IAA17 (PDB:2muk.1) was used as the reference template for protein tertiary structure analysis, and the protein sequence similarity between the two was 47.52% (Fig. 4A). *PmaARF15* contains 949 amino acids, the highest of which is serine (101; 10.6%), containing 100 positively charged amino acid residues and 120 negatively charged amino acid residues. The isoelectric point is 5.77, and it is an acidic protein (negatively charged) with the molecular structure formula C_4599_H_7209_N_1305_O_1453_S_40_, molecular weight 105313.10 U, unstable protein. No transmembrane movement of the protein was analyzed by TMHMM. This protein has phosphorylation sites, including 71 serines (Ser), 34 threonines (Thr) and 8 tyrosines (Tyr), all of which are potential phosphorylation sites for protein kinases. The protein was predicted to be a nonsecretory protein by signALP-4.1. WoLF PSORT analysis indicated that this protein was most likely to exist in the nucleus. Secondary structure of protein of NovoPro showed that the protein had 46 α-helices and 34 β-folds (Fig. 3B). Crystal Structure of the ARF5 oligomerization domain (PDB: 4chk.4) was used as the reference template for protein tertiary structure analysis, and the protein sequence similarity between the two was 47.52% (Fig. 4B).

**Fig. 5:**
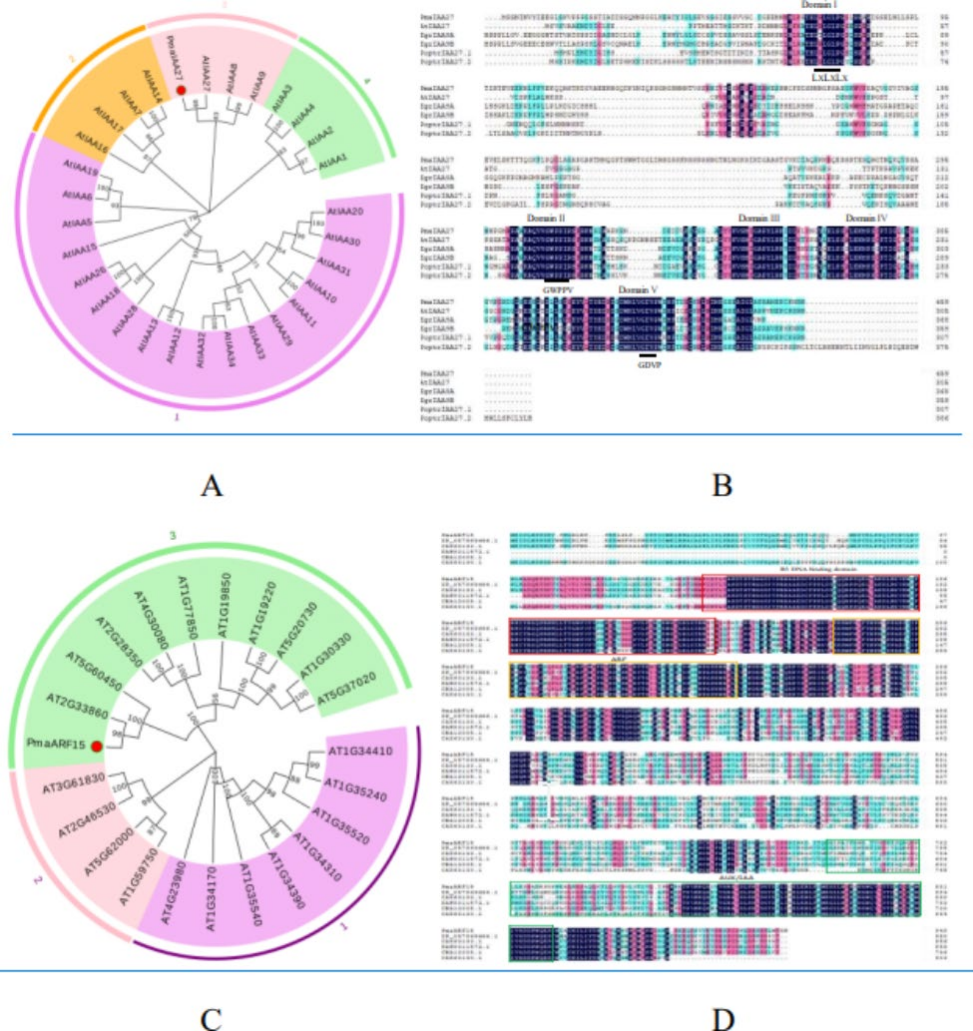
Phylogenetic analysis and sequence alignment of *PmaIAA27* and *PmaARF15* with homologue genes. (**A**) Phylogenetic analysis of *PmaIAA27* and 29 Aux/IAA proteins in Arabidopsis. The tree was produced using MEGA 11 Bootstrap values from 1000 replicates are specified at each branch. Pma(*P.massoniana*), At( *Arabidopsis thaliana*). Red circles, represent genes from *P.massoniana.* (**B**) Alignment of amino acid sequence of Aux/IAA proteins from several plant species. Egr(*Eucalyptus grandis*), Poptr (*Populus trichocarpa*). Amino acid sequences were aligned by DNAMAN. The domain I-IV and motifs were marked with black line. (**C**) Phylogenetic analysis of *PmaARF15* and 22 ARF proteins in Arabidopsis. (**D**) Alignment of amino acid sequence of ARF proteins from several plant species. (Pma; XP_057869666.1,*Cryptomeria japonica*); CAX63133.1, *Ginkgo biloba*; KAH9311572.1, *Taxus chinensis*; CBA12005.1, *Cycas rumphii*; CAX63130.1,*Ephedra distachya*). Domain B3 DNA binding, ARF, AUX/IAA are represented by red, orange and blue boxes, respectively

Furthermore, we performed phylogenetic analysis with 29 Aux/IAA and 22 ARF proteins from Arabidopsis. It was indicated that the deduced Aux/IAA protein is clustered together with AtIAA27 and ARF protein is clustered together with AT2G33860 (Fig. 5A.C). Additionally, we downloaded the sequences of five IAA and ARF proteins from different plant species and multiple sequence alignment was performed. The domains of *PmaIAA27* proteins were further divided into Domain I-V. The domains of *PmaARF15* proteins were further divided into Domain B3 DNA binding, ARF, AUX/IAA (Fig. 5B.D).

### Characteristics of the transgenic tobacco *PmaIAA27*

**Fig. 6:**
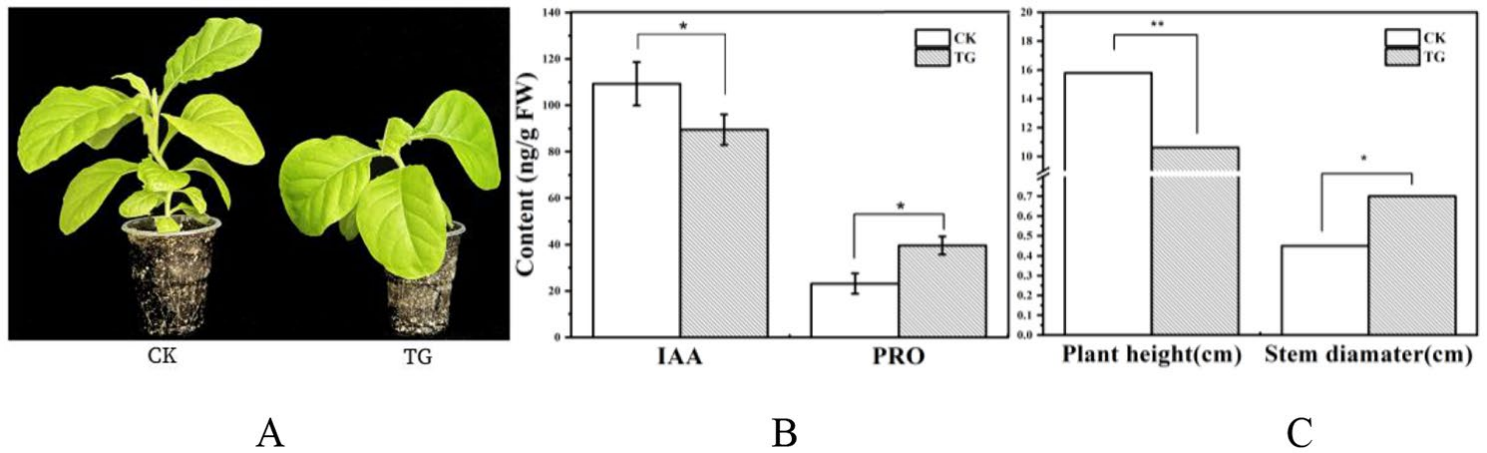
*Phenotype of PmaIAA27 transgenic tobacco* (**A**) and *leaf IAA*, *PRO contents* (**B**) and Plant height and Stem diameter (**C**). * indicates that the difference between different temperatures at the same drought level is significant at 0.05 level; ** represents a significant difference at 0.01 level. CK indicates non-transgenic normal plants and TG indicates transgenic plants

The *PmaIAA27* gene was introduced into SR1 tobacco via Rhizobium radiobacter-mediated transformation. Kanamycin resistance screening confirmed the successful integration of the target gene into Rhizobium radiobacter. Hygromycin selection was employed to identify positive transgenic plants. F2 seeds from these positive plants were cultivated in soil for 30 days, resulting in a total of 17 transgenic tobacco plants (Additional file 1: Fig. 7). Among them, the transgenic tobacco with the highest expression level was compared to the control (CK) (Fig. 6A). Compared to control plants, TG plants exhibited significant reductions in height and internode length, while their stem diameter ratio increased significantly (Fig. 6C). Analysis of IAA content revealed a significant decrease in TG plants, consistent with their phenotypic characteristics. Additionally, proline content in leaves of TG plants showed a significant increase compared to controls (Fig. 6B), suggesting that overexpression of the *PmaIAA27* gene may enhance drought resistance in plants.

### Yeast double hybrid assay of *PamIAA27* and *PamARF15* genes

The results of *PmaARF15* plasmid toxicity assays and autoactivation assays are presented in Additional file 1: Fig. 1. These findings demonstrate that successful transformation of PGBKT7-*PmaARF15* into Y2HGold did not result in any observed toxicity to the host, as indicated by growth in DDO medium. Furthermore, growth in TDO medium confirmed that the PGBKT7-*PmaARF15* protein was capable of activating the expression of the yeast cell reporter gene His3; however, it failed to grow in TDO/3’AT (10mM), suggesting that 3’ AT (10mM) could inhibit the expression of PGBKT7-*PmaARF15* protein within yeast cells. Notably, no growth was observed in QDO medium, indicating an inability for the PGBKT7-*PmaARF15* protein to activate the expression of ADE2 reporter gene within yeast cells. Consequently, subsequent validation tests were performed using TDO/3’AT (10mM) and QDO.

**Fig. 7:**
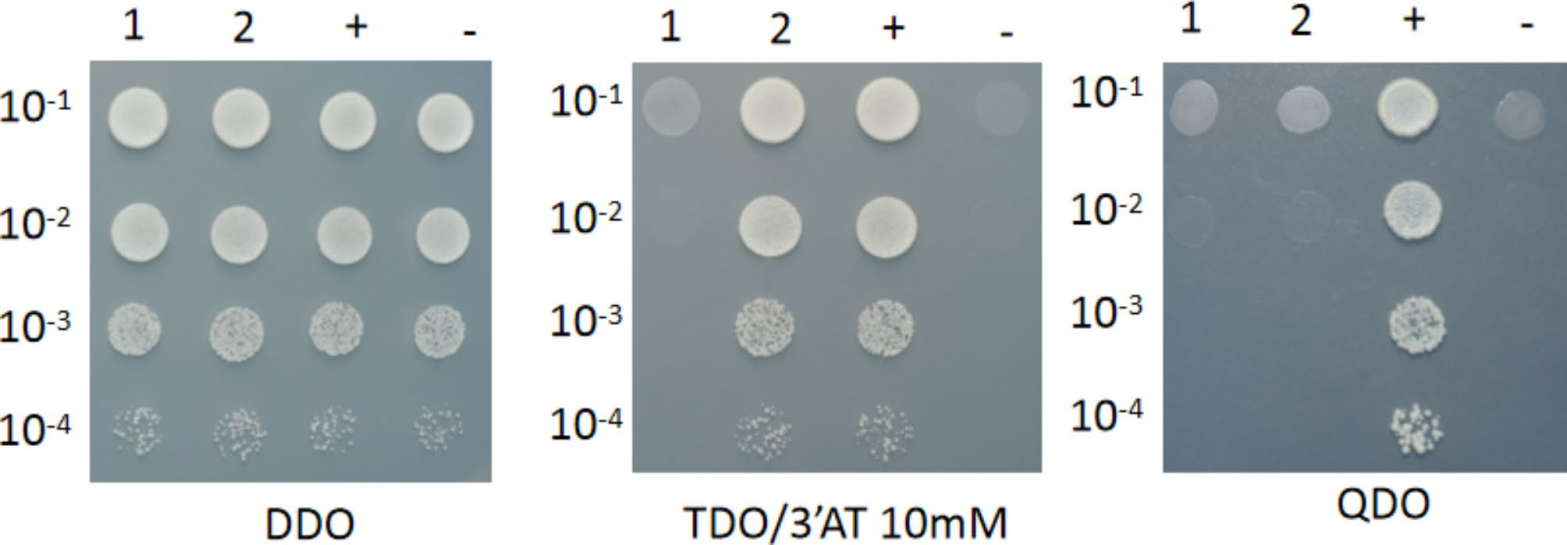
Dilution verification picture. 1: PGBKT7-*PmaARF15* + PGADT7. 2:PGBKT7-*PmaARF 15* + PGADT7-*PmaIAA27.* +: pGBKT7-53 + pGADT7-T. -: pGBKT7-lam + pGADT7-T. DDO: SD/-Leu/-Trp. TDO: SD/-Leu/-Trp/-His. 3’AT 10mM: 10mM 3-amino-1,2,4-triazole. QDO: SD/-Leu/-Trp/-His/-Ade

Yeast cells co-transformed with PGADT7-*PmaIAA27* and PGBKT7-*PmaARF15*, along with positive and negative controls, were analyzed as shown in Additional file 1: Fig. 2. Combined with dilution validation results (Fig. 7) analysis found that: the results of control experiments were consistent with the expected outcomes, indicating the suitability of this system for yeast hybridization verification. Co-transformed yeast cells carrying PGBKT7-*PmaARF15* + PGADT7-*PmaIAA27* exhibited growth on DDO medium and TDO/3’AT (10mM) medium but not on QDO medium, confirming activation of the reporter gene His3 expression. In conclusion, an in vitro interaction between *PmaARF15* and *PmaIAA27* was observed.

## Discussion

It has been reported that Aux/IAA family genes play a crucial role in regulating plant stress [28]. Few studies have reported that Aux/IAA genes can improve the stress resistance of plants [22, 29, 30]. In *Arabidopsis thaliana*, Aux/IAA proteins IAA-5, -6, and − 19 are responsible for stomatal modulation and drought stress responses by regulating glucosinolates levels [31]. The expression of the *OsIAA* gene elevated significantly in rice under stress [32, 33]. Overexpression of *OsIAA6* significantly enhanced drought tolerance in rice [34]. Rice *OsIAA20* also showed an upregulation trend under stress [35].

In this study, it was found that exogenous irrigated auxin significantly decreased *PmaIAA27* gene expression and increased *PmaARF15* gene expression in Masson pine seedlings, which may be due to the increase in endogenous auxin content in Masson pine seedlings, thereby inhibiting and promoting the expression of *PmaIAA27* and *PmaARF15* genes, respectively. To further study the function of the *PmaIAA27* gene, transgenic tobacco was transformed with *the PmaIAA27* gene of Masson pine. The results showed that the auxin content of tobacco transformed with the *PmaIAA27* gene was remarkably lower than that of control plants. The reason for this phenomenon is likely to be that the overexpression of *PmaIAA27* gene causes the increase of IAA transcription suppressor, which further binds to the target promoter of ARF, inhibits the expression of auxin response gene, and ultimately leads to the decrease of auxin content. Transgenic tobacco showed a trend of growth reduction and significant dwarfing. In potato studies, it was found that the increased expression of miR160a5p led to the inhibition of the target gene *ARF16* gene, and the transgenic plants showed increased proline level and enhanced stress resistance [36]. This is consistent with the results of this study, which showed that overexpression of *PmaIAA27* gene caused the increase of proline in transgenic plants. It is likely that overexpression indirectly changed the expression of genes encoding proline synthesis and increased the accumulation of proline in transgenic plants. The proline content plays a vital role in regulating osmotic stress homeostasis in plants. The increase in proline content allows plant cells to maintain water and improve stress resistance [37]. Therefore, overexpression of the *PmaIAA27* gene of Masson pine may be helpful to improve the drought resistance of plants. It was found that overexpression of the rice *OsIAA18* gene enhanced salt tolerance and permeability in *Arabidopsis thaliana* [38]. In addition, the *VvIAA18* gene from grapes was cloned from PN40024 (grapevine cultivar), and tobacco plants overexpressing *VvIAA18* showed improved salt tolerance [38, 39]. Rice plants overexpressing *OsIAA6/OsIAA20* showed stronger salt tolerance and drought tolerance, which is in line with the findings of this study [34, 35]. However, Aux/IAA genes belong to an extensive gene family, which has different functions and can modulate a variety of hormone signaling pathways, including salicylic acid, jasmonic acid, brassinolide steroids, and ethylene [33, 40–42]. This study only preliminarily confirmed the function of *PmaIAA27* gene, and the functions of other family members need to be further explored.

ARFs genes not only participate in plant growth and development, but also play an important role in regulating plant tolerance to biotic and abiotic stresses. Relevant studies found that silencing BpARF1 gene by RNA interference increased the content of ascorbic acid and proline, while overexpressing plants showed opposite physiological changes [43]. *SaARF5*, *SaARF10* and *SaARF16* of S.album (*Santalum album* L.) were also found to be overexpressed under drought stress [44]. In addition, increasing the level of Mdm-miR160 targeted to reduce the content of *MdARF17* also improved the drought tolerance of plants [45]. Knockout of *SlARF2* in tomato resulted in higher levels of soluble sugar and proline [46]. Therefore, ARFs gene suppression can effectively improve the drought resistance of plants. This study also found that, through the overexpression of Masson Pine *PmaIAA27* genes, tobacco show that the proline content, investigate its reason, maybe it’s because *PmaIAA27* gene expression, suppresses the ARF gene expression, also need to be further in-depth study.

Bioinformatics analysis of *PmaIAA27* and *PmaARF15* genes showed that these two genes both contain complete domains of the Aux/IAA and ARF families, respectively. Aux/IAA domains III and IV interact with the ARF protein PB1 to form dimers [47]. The PB1 domain of *PmaARF15* gene is concentrated in the 821–905 bp interval. Therefore, we intercepted the PB1 structural region of *PmaARF15* gene and *PmaIAA27* gene for the yeast two-hybrid test. The interaction between PB1 domain of *PmaARF15* gene and *PmaIAA27* gene was successfully verified in this study, which was consistent with previous results [48]. However, this study only confirmed the interaction between the PB1 domain of *PmaARF15* gene and *PmaIAA27*, while the functions of other domains need to be further explored.

## Conclusions

In summary, we investigated the function of the *PmaIAA27* gene and its interaction with the *PmaARF15* gene. The results demonstrated that exogenous auxin irrigation down-regulated the expression of the *PmaIAA27* gene and significantly up-regulated the expression of the PmaARF15 gene in Masson Pine seedlings. Compared to control plants, transgenic tobacco plants carrying the *PmaIAA27* gene exhibited a significant decrease in auxin content but a significant increase in proline content, suggesting that overexpression of *PmaIAA27* may enhance drought resistance in Masson pine. To further validate their interaction, we conducted bioinformatics analysis and yeast two-hybrid experiments on these genes. The findings revealed a specific interaction between the PB1 structural region of *PmaARF15* and the *PmaIAA27* gene. These discoveries provide valuable insights into understanding stress tolerance mechanisms in Masson pine and expand our repertoire of candidate genes for activating abiotic stress tolerance improvement. Given the complexity of auxin-related signaling pathways, it is imperative to systematically evaluate target genes involved in ARF-mediated auxin signaling to comprehensively comprehend development and environmental adaptation processes in Masson pine.

## Materials and methods

### The flow chart

**Fig. 8:**
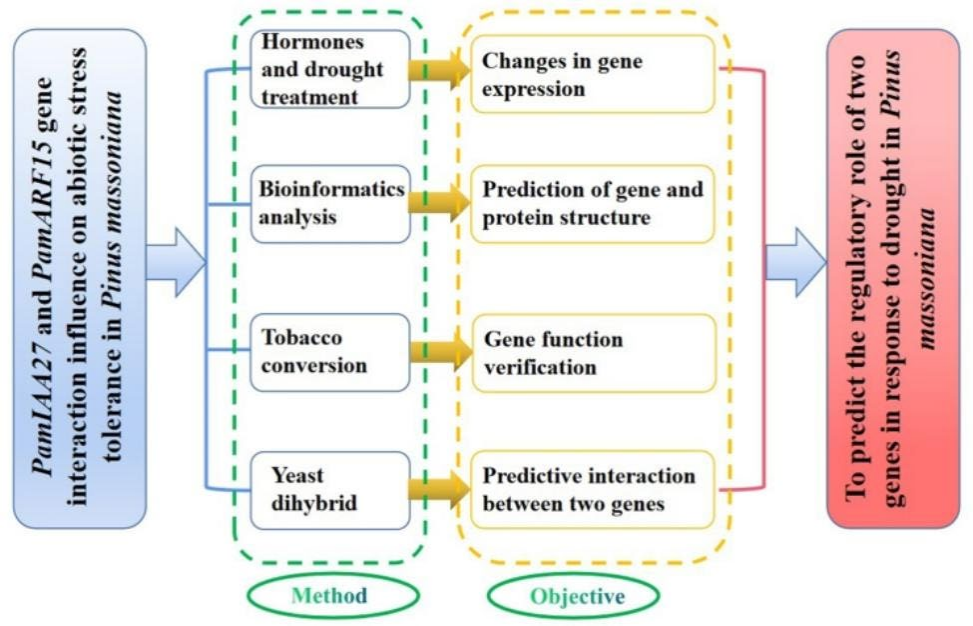
Test flow chart

To predict the roles of *PmaARF15* and *PmaIAA27* in the response to drought in *P. massoniana*, we analyzed the two genes using hormone drought treatment in Masson pine seedlings, bioinformatics analysis of the two genes, transtobacco and yeast double hybrid, respectively. The specific flow chart of the test is shown in Fig. 8.

### Plant materials and growth conditions

**Fig. 9:**
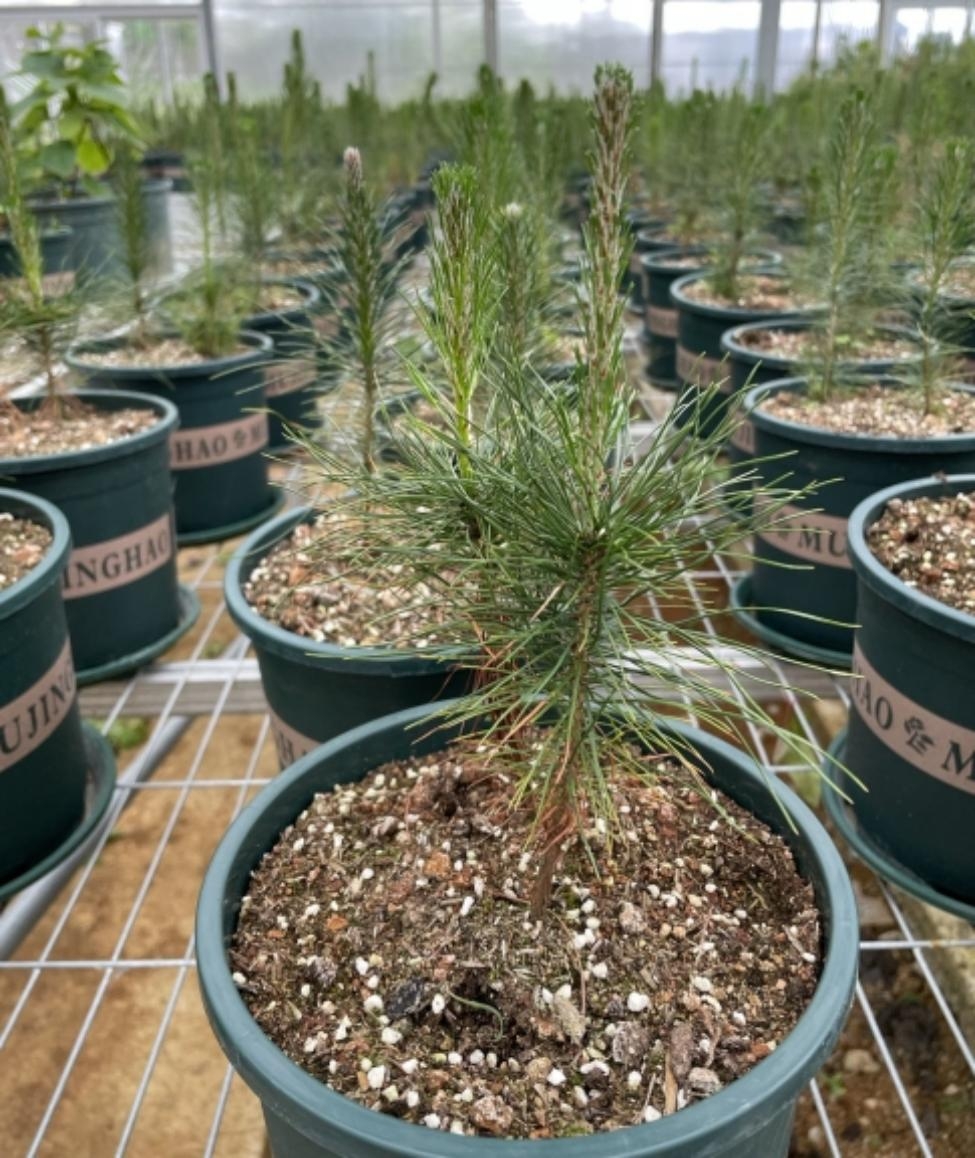
Masson pine seedlings used in the experiment

One-year-old Masson pine seedlings from Dushan County, Guizhou Province, were transplanted onto flowerpots in the nursery of Guizhou University. The flowerpot had an upper diameter of 300 mm, a lower diameter of 200 mm, and a height of 250 mm. Yellow soil was collected from the Masson pine forest, and the same amount of soil was placed in each basin. During this period, normal growth and management lasted for 5 months. Finally, 40 seedlings at the same growth stage were selected for the experiment (Fig. 9).

### Drought stress treatment and hormone treatment

Because of Masson pine mainly concentrated in the fast growing season in March to September. Therefore, the official start of the trial was on June 10, 2022. The drought (D) group and drought hormone (DH) group were designed, and 20 basins of Masson pine seedlings with consistent growth were randomly selected in each group. The seedlings in the D group were fully watered for the first 7 days, while those in the DH group received the same volume of 1 mM IAA for the first 7 days and then sustained drought. The soil water content was recorded daily, and the leaves were sampled at 30-35% maximum field capacity (FC) (severe drought, SD), 40-45% FC (moderate drought, MD), 55-60% FC (light drought, LD), and 75-80% FC (control, CK). All sample were frozen in liquid nitrogen and immediately placed at -80 °C.

### RNA extraction and cDNA synthesis

Young tobacco leaves were accurately weighed (0.1 g) and fully ground in liquid nitrogen, following the strict operating instructions of the RNAprep Pure Plant Plus Kit (Polysaccharides Polyphenolics-rich, DP441, TIAN GEN, Beijing, China). The purity and concentration of RNA were determined using a microspectrophotometer. Qualified RNA was used for cDNA synthesis, which was performed strictly according to the instructions for use of the RNA PCR Kit v2.1 (TaKaRa, Dalian, China), employing AMV Reverse Transcriptase XL as the enzyme. Finally, the concentration and purity of cDNA were determined using a trace spectrophotometer.

### Real-time quantitative PCR (RT‒qPCR) analysis

RT‒qPCR was conducted on the ABI 7500 Detection System (Applied Biosystems, USA) using an SYBR GREEN PCR Master Kit (TaKaRa). Primer 5.0 software was used to design quantitative Real-time PCR primers for genes. The specific primers were shown in Additional file 1: Table 2. The *UBC* gene was used as an internal control [49]. The 2^−ΔΔCT^ method [50] was utilized to calculate mean expression levels and standard deviation (SD).

### Bioinformatics analysis

The full-length sequences of the target gene were obtained by comparing the known target gene sequence with the complete transcriptome of the third generation of Masson pine (NCBI: Three-generation transcriptome (PRJNA970047)). Open reading frames were identified using NCBI ORF Finder (https://www.ncbi.nlm.nih.gov/orffinder/), and BLAST analysis (https://blast.ncbi.nlm.nih.gov/Blast.cgi) was performed to assess homology, conserved regions, and active sites in protein sequences. ProtParam on the ExPaSy website (https://web.expasy.org/protparam/) was utilized to evaluate amino acid composition, isoelectric point, and hydrophilicity. NetPhos online prediction (NetPhos 3.1-DTU Health Tech- Bioinformatic Services) was employed for protein phosphorylation site prediction. Subcellular localization of proteins was estimated using WoLF PSORT online (https://wolfpsort.hgc.jp/). Secondary structure analysis of proteins was conducted through NovoPro website’s DSSP tool (https://www.novopro.cn/tools/dssp.html). Swiss-model software and the Phyre2 tool (SWISS-MODEL Interactive Workspace (expasy.org)) were used to characterize the homologous tertiary structure of amino acid sequences. Protein alignment was performed using Clustal W in MEGA version 5.0, followed by construction of a neighbor-joining phylogenetic tree employing bootstrap method with 1,000 replications, p-distance calculation, and pairwise deletion.

### Gene cloning

SnapGene software was used to design gene ORF region span primers as *PmaIAA27*-F1: 5’-3’ ATGTCGTCTAATATTAATGTTTATATCGAGG; *PmaIAA27*-R1: 5’-3’ CTAGTTCCTGTTTTTAC ATTTCTCCATG. The ORF region of *PmaIAA27* was subjected to PCR amplification using *PmaIAA27*-F1 and *PmaIAA27*-R1 as primers and cDNA as a template. Reaction system: 10 µL mixture, 0.5 µL primer each, 1 µL cDNA, and 8 µL ddH_2_O. Reaction procedure: 5 min at 94 °C, 30 s at 94 °C, 30 s at 57 °C, 10 min at 72 °C. After purification and recovery with Gel Purification Recovery Kit, the target PCR product was transformed into *E. coli.* DH5α receptor cells after ligation with the pMD19-T cloning vector. Amp-resistant single colonies were selected for colony PCR identification, and the positive clones were sequenced by Sangong Bioengineering (Shanghai Co., Ltd.).

### Construction of expression vectors

SnapGene was employed to design specific primers for the ORF region of the target gene: *PmaIAA27*-F2: 5’-3’GGGGTACCATGTCGTCTAATATTAATGTTTATATCGAGG and *PmaIAA27*- R2: CG GAATTCCTAGTTCCTGTTTTTACATTTCTCCATG. The 5’ end of the upstream and downstream primers were added with KpnI and EcoRI restriction sites and protective bases, respectively, for vector construction. The ORF region of *PmaIAA27* gene was subjected to PCR amplification using the recombinant cloning vector pMD19-*PmIAA27* as a template and *PmaIAA27*-F2 and *PmaIAA27*-R2 as primers. The reaction system and procedure were similar to those described in 2.6. The amplified product was recovered by 1% agarose gel electrophoresis. The overexpression vector pCAMBIA1301 (Additional file 1: Fig. 5) was digested by the restriction endonucleases KpnI and EcoRI. The enzyme digestion system was as follows: 3 µL 10× Buffer, 1 µL KpnI, 1 µL EcoRI, 15 µL pCAMBIA1301 carrier, 10 µL ddH_2_O, total volume 30 µL. Reaction conditions: 37 °C, 2 h; 4 °C. The products were analyzed by 1% agarose gel electrophoresis and then recovered. The recombinant vector pCAMBIA1301-*PmaIAA27* was constructed by T4 DNA ligase from TaKaRa Company. Reaction system: 1 µl pCAMBIA1301 carrier, 2 µl *PmaIAA27*, 2 µL Infusion Mixture, 5 µL ddH_2_O, 10 µL total volume. Reaction conditions: 50 °C, 30 min; 4 °C, stored. After transforming the recombinant vector into E. coli. DH5α using the heat excitation method, Amp-resistant single colonies were picked for colony PCR identification. Verification of the positive clones was conducted by sequencing. The recombinant plant overexpression vector pCAMBIA1301-*PmaIAA27* was transformed into *Rhizobium radiobacter* GV3101 using the freeze-thaw approach, and positive clones were detected by PCR using *PmaIAA27*-F2 and *PmaIAA27*-R2 as primers.

### Genetic transformation of tobacco

The sterile tobacco leaves (SR1) were cut into small cubes of 0.5 cm^2^ and placed in the prepared *Rhizobium radiobacter* for 10 min. The infested or infected leaves were placed on sterile filter paper and blotted dry. The leaves were tiled downward on MS co-culture medium and cultured at 28 °C for 5 d in the dark. The co-cultured tobacco leaves were transferred to MS bud induction medium (MS + 1.5 mg/L 6-BA + 0.1 mg/L NAA + 50 mg/L HYG + 200 mg/L CTX) and incubated at 28 °C in the light. The medium was changed every 15 d. When adventitious buds grew approximately 1–2 cm in the tobacco leaf discs, the differentiated resistant buds were cut down and transferred into MS rooting medium (1/2MS + 0.01 mg/L NAA + 50 mg/L HYG + 200 mg/L CTX) to promote their rooting growth. When the root system was fully developed, the seedlings were moved into the soil for culture and routine management. The removed resistant transgenic tobacco was numbered, RNA was extracted from the leaves, and cDNA was reverse transcribed. The transgenic plants were detected by PCR using *PmaIAA27*-F1 and *PmaIAA27*-R1 as primers.

### Yeast two-hybrid validation

Toxicity and self-activation assay was performed for *PmaARF15* recombinant plasmid. Briefly, the carrier DNA was heated at 100℃ for 5 min and placed on ice for 2 min, and this step was repeated once. The transformation system was conFig.d, 50 µL Y2H Gold yeast responsive cells + 5 µL Carrier DNA + 100 ng *PmaARF15* recombinant plasmid + 100 ng PGADT7 empty vector. Then, 500 µl PEG/LiAc was slowly added. The mixture was heated at 30℃ water bath for 30 min, and then added with 20 µl DMSO. The supernatant was removed by the water bath at 42 °C for 15 min, followed by centrifugation (800 ×g, 1 min), 800 µl of YPDA for resuspension, 150 rpm at 30 °C for 1.5 h, and centrifugation (800 ×g, 5 min). After discarding the supernatant and 1 mL of 0.9% NaCl for resuspension, the culture was inverted on a 150 µL coated plate at 30℃ for 5 d. Colony diameter and color were observed. For cotransformation validation, the test procedures were similar to those described in above. The experimental and control groups are listed in Additional file 1: Table 2.

Dilution point seeding. Single colonies were picked from the self-activated, cotransformed experimental group and cotransformed negative control group coated with plate DDO, respectively, inoculated in 5 ml DDO liquid medium, followed by 250 rpm incubation at 30℃ for 16 h. Then, 300 µL of the bacterial solution was placed in 50 ml DDO, followed by 250 rpm incubation 30℃ until reaching an OD600 value of 0.6. Four 0.2 ml PCR tubes were selected for both control and experimental groups, added with 90% NaCl, and numbered as A, B, C, and D. Then, 10 µL of the above shaken bacterial solution was placed into each tube, mixed well, and placed onto a plate. DDO, TDO, and QDO were incubated at 30 °C for 3 d, and the growth of bacterial spots was observed.

### Statistical analysis

All assays were repeated at least 3 times, and the data were expressed as mean ± SD. Statistical differences were measured using Duncan’s test at the p = 0.05 level. Data processing was conducted with Microsoft Excel 2010. Line and column charts were drawn by Origin v8.5.

### Electronic supplementary material

Below is the link to the electronic supplementary material.

Supplementary Material 1

## Data Availability

The data used and/or analyzed during the current study are available from the corresponding author on reasonable request. The datasets generated and/or analyzed during the current study are available in the Sequence Read Archive, under the Gen Bank Transcriptome Shotgun Assembly (TSA) database (GFHB00000000) and Three-generation transcriptome (PRJNA970047) at NCBI.
