# Correction: Effects of *PmaIAA27* and *PmaARF15* genes on drought stress tolerance in *pinus massoniana*

**DOI:** 10.1186/s12870-023-04544-w

**Published:** 2023-10-25

**Authors:** Liangliang Li, Yan Li, Wenxuan Quan, Guijie Ding

**Affiliations:** 1 Forest Resources and Environment Research Center, Key Laboratory of Forest Cultivation in Plateau Mountain of Guizhou Province, College of Forestry, Guizhou University, Guiyang, 550001 China; 2 Institute of Mountain Resources of Guizhou Province, Guiyang, 550001 China

**Correction: *BMC Plant Biol*** **23, 478 (2023)**

10.1186/s12870-023-04498-z

Following publication of the original article [1], mouse cursors were mistakenly included and appear to be visible in Figs. 2, 3 and 4, and 6.

The corrected figures are provided below:

**Fig. 2:**
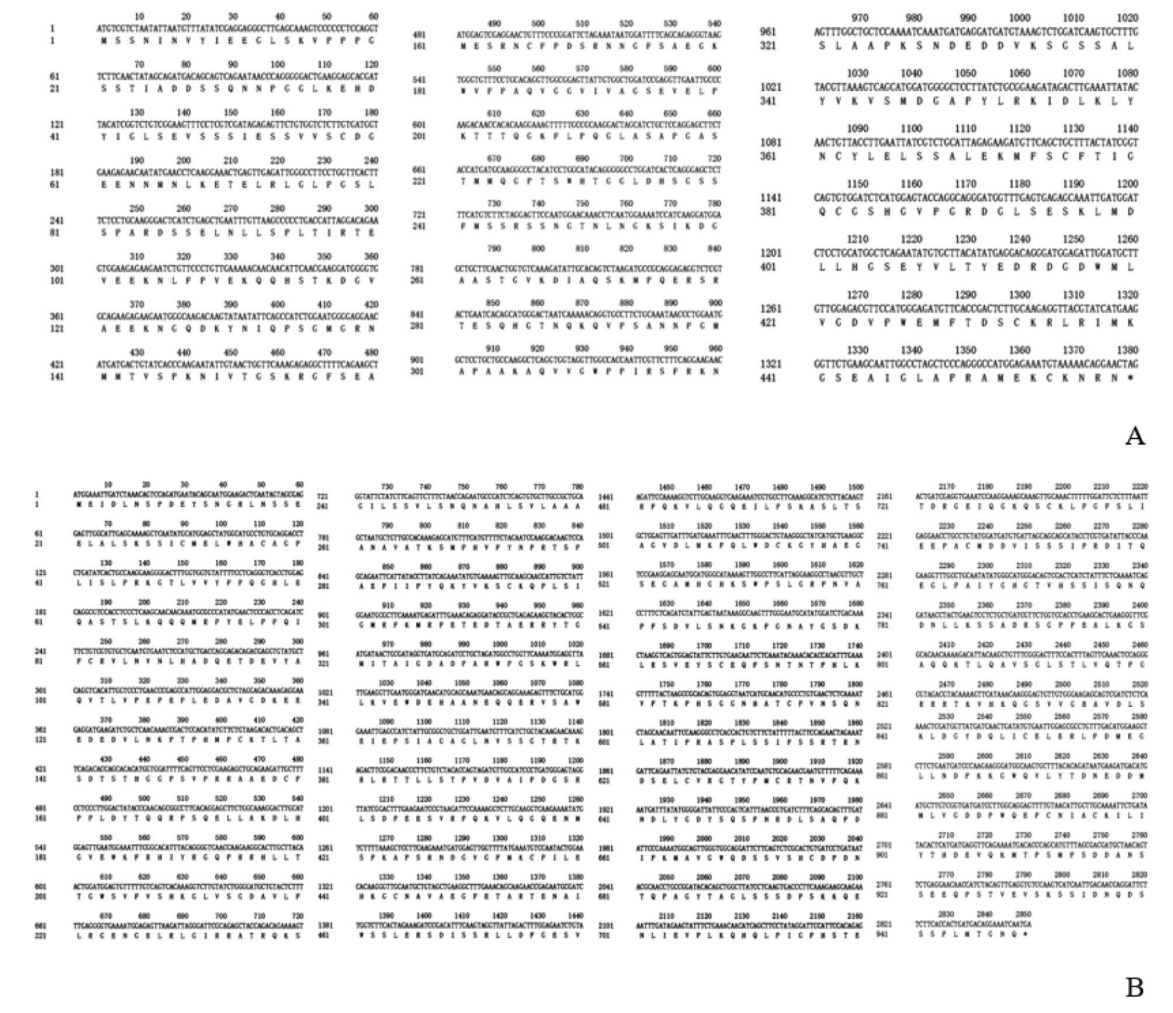
Coding sequences and derived amino acid sequences of *PmaIAA27* (**A**) and *PmaARF15* (**B**)

**Fig. 3:**
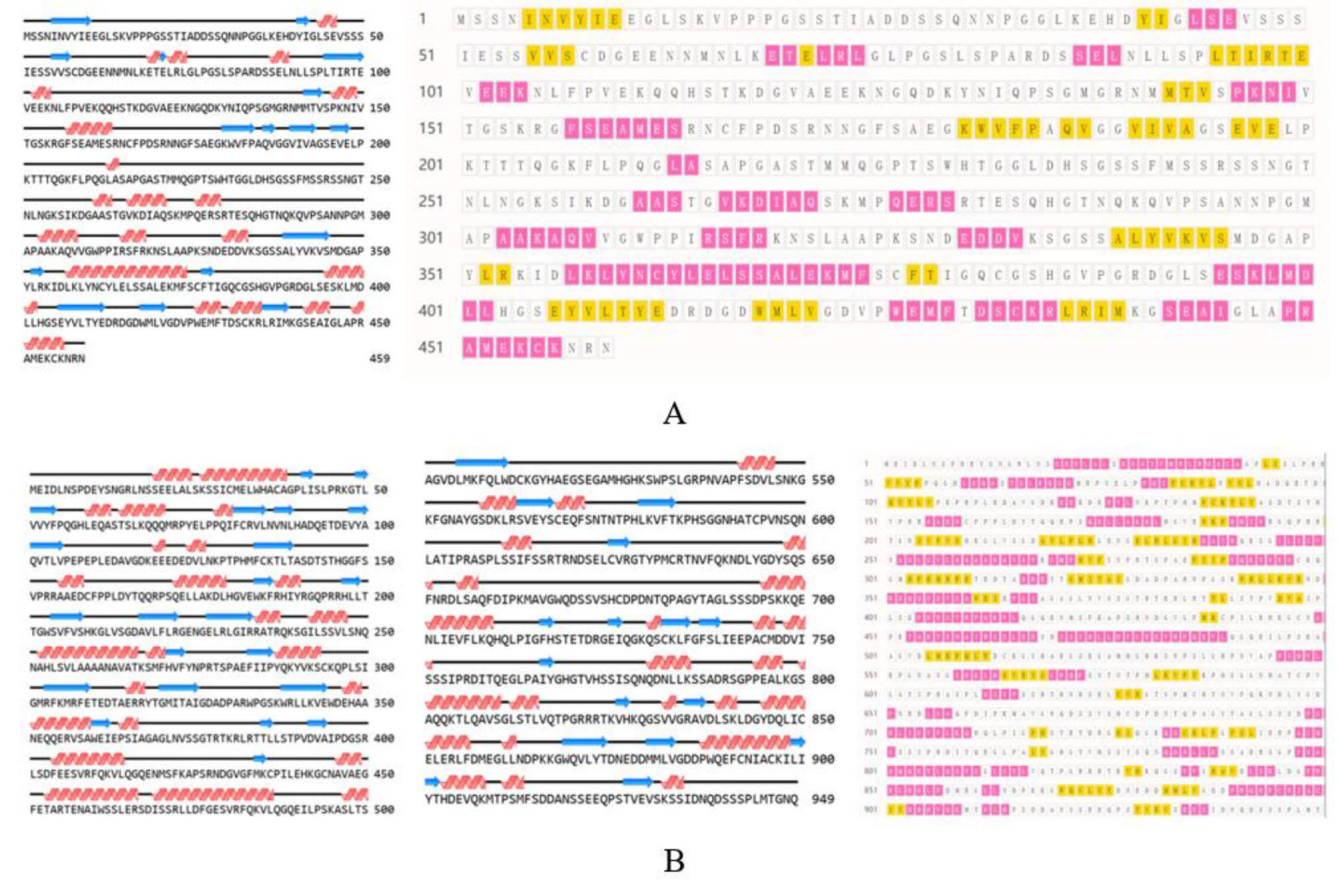
Analysis of the secondary structure of the proteins encoded by *PmaIAA27* (**A**) and *PmaARF15* (**B**). Red represents α-helix, yellow and blue arrows represents β-sheet

**Fig. 4:**
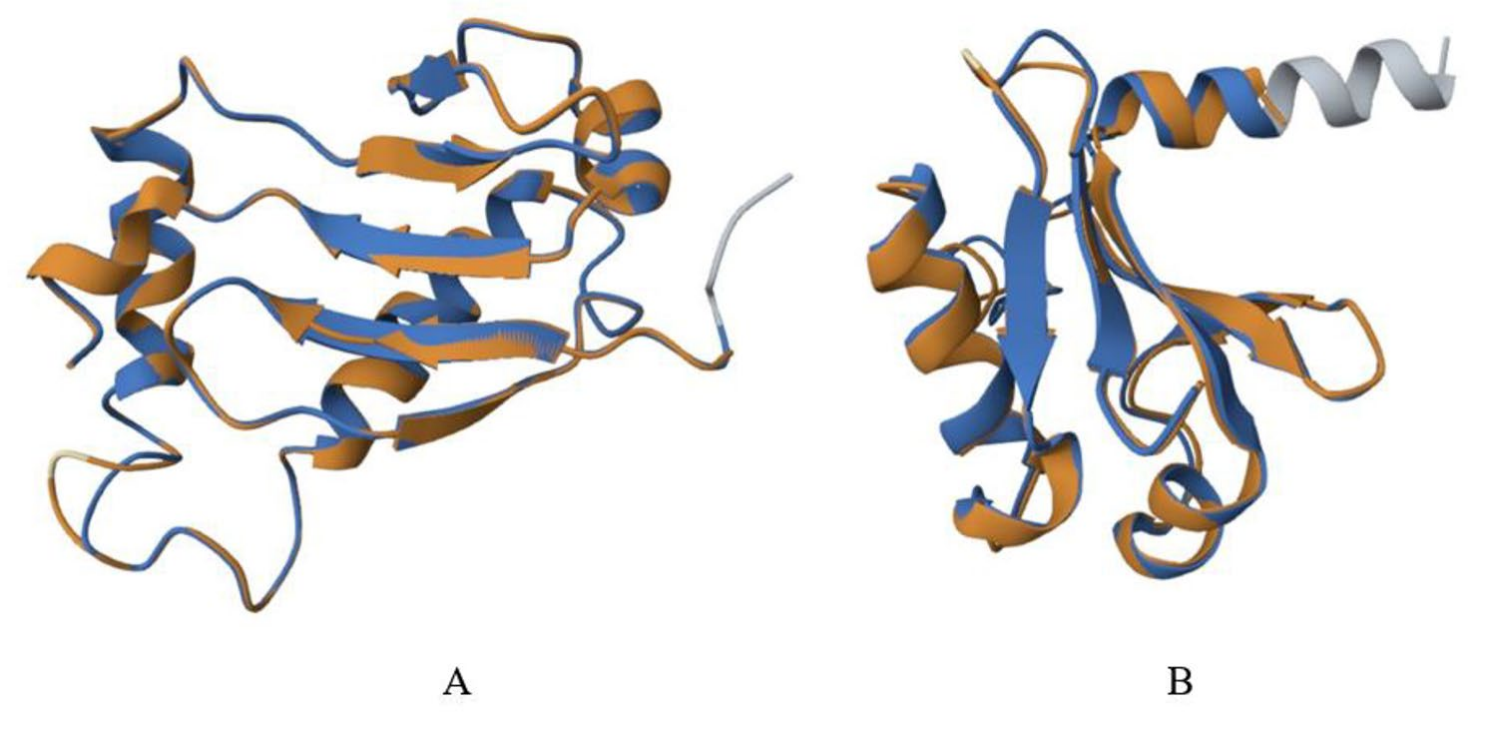
*PmaIAA27* (**A**) and *PmaARF15* (**B**) encoding protein tertiary structure homology modeling. Orange and blue are aligned amino acid residues for the target and template proteins, respectively, and all other colors are unaligned amino acid residues

**Fig. 6:**
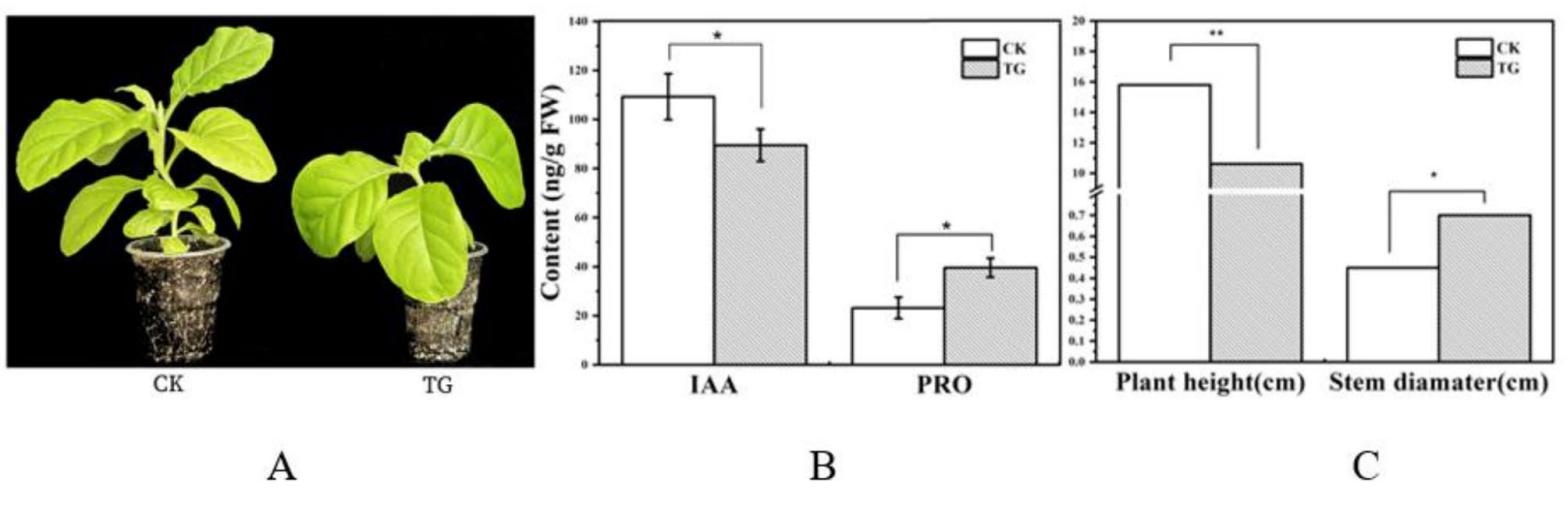
*Phenotype of PmaIAA27 transgenic tobacco* (**A**) and *leaf IAA, PRO contents* (**B**) and Plant height and Stem diameter (**C**). * indicates that the difference between different temperatures at the same drought level is significant at 0.05 level; ** represents a significant difference at 0.01 level. CK indicates non-transgenic normal plants and TG indicates transgenic plants

The correction does not affect the overall result or conclusion of the article.
