# Supplementary material for: Effects of PmaIAA27 and PmaARF15 genes on drought stress tolerance in pinus massoniana
Source: BMC Plant Biol. 2023 Oct 9;23:478. doi: 10.1186/s12870-023-04498-z (PMC10561430; doi:10.1186/s12870-023-04498-z)
Supplement: Supplementary file 1 — Supplementary Material 1 [file 12870_2023_4498_MOESM1_ESM.docx]

**Additional file**

**Figure**


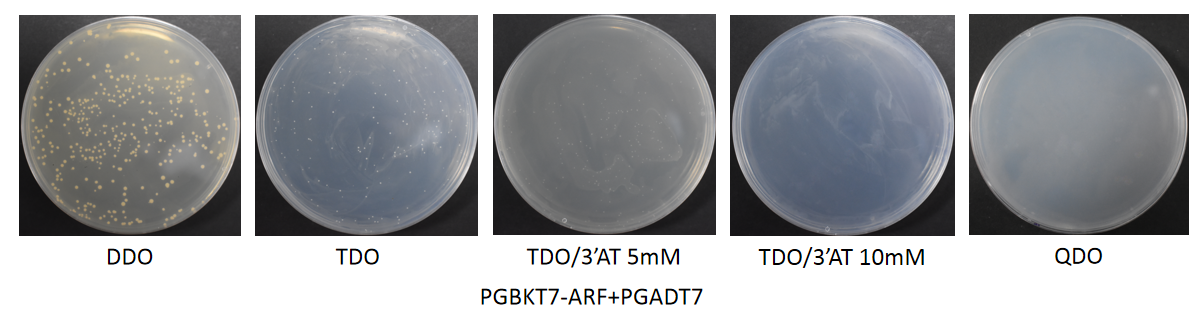


Fig. 1 PGBKT7-*PmaARF15*+PGADT7. DDO: SD/-Leu/-Trp. TDO: SD/-Leu/-Trp/-His. 3’AT 10mM: 10mM 3-amino-1,2,4-triazole. 3’AT 5mM: 5mM 3-amino-1,2,4-triazole.QDO: SD/-Leu/-Trp/-His/-Ade.

*
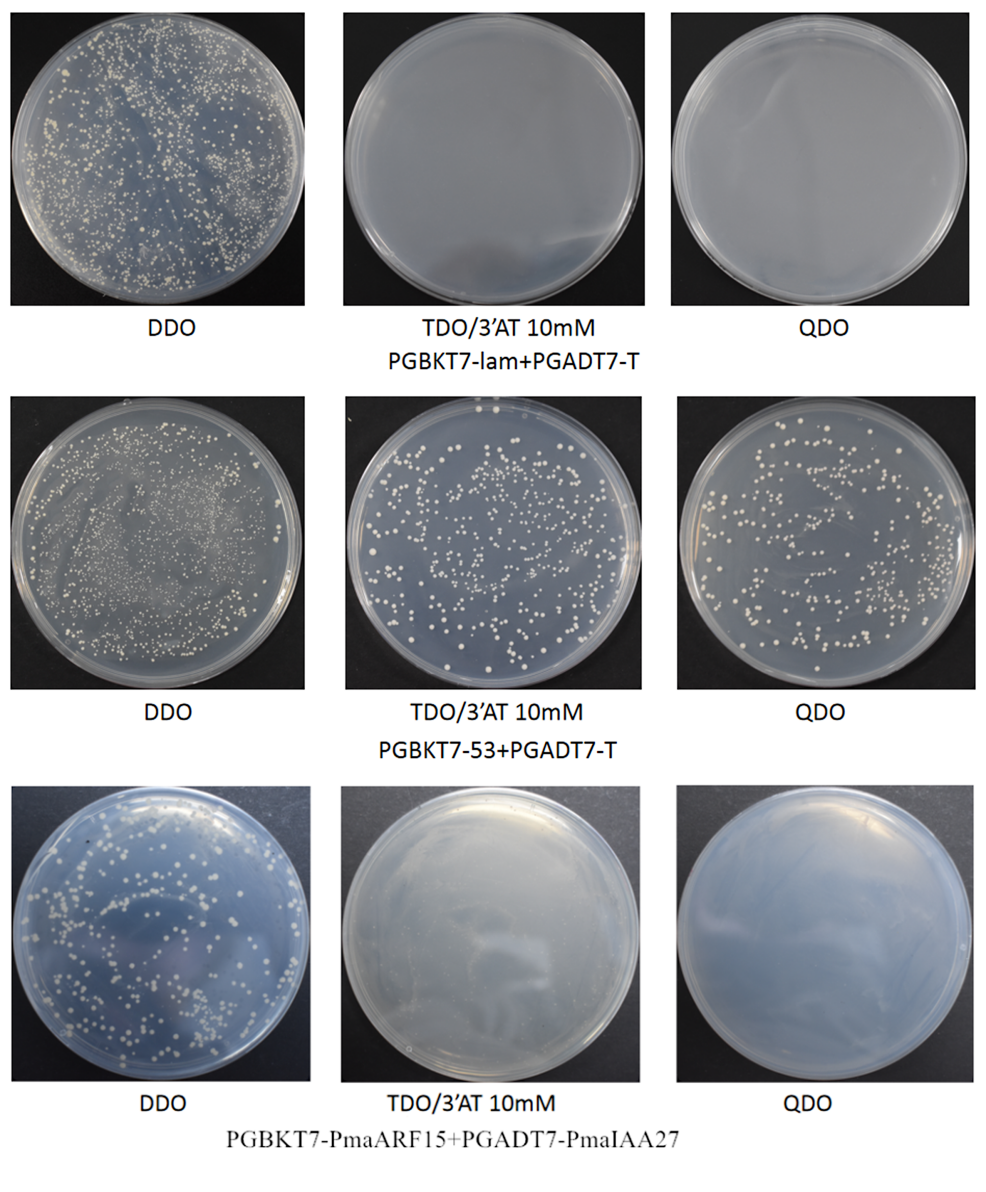
*

Fig. 2 Cotransformation of Y2H gold yeast. Note: The first row shows the experimental group conversion; the second row shows PGBKT7-LAM and PGADT7-T (negative control); and the third row shows PGBKT7-53 and PGADT7-T (positive control).

*
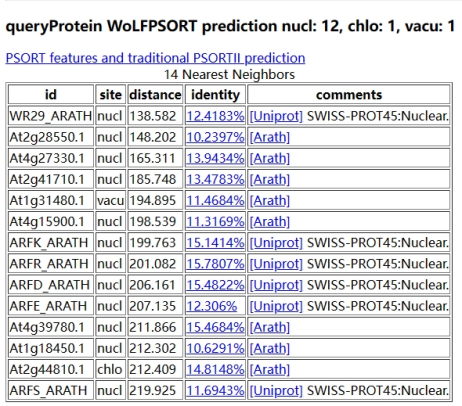

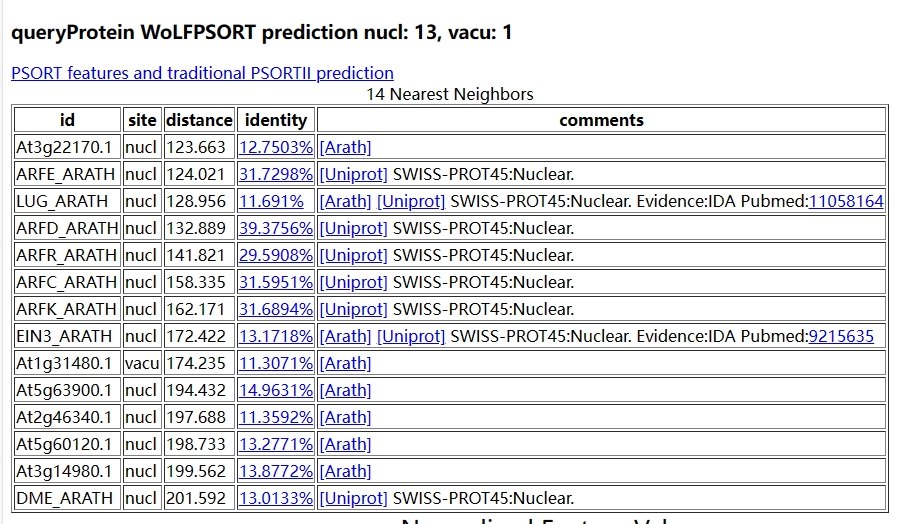
*

A B

Fig. 3 WoLF PSORT results. A: *PmaIAA27*. B：*PmaARF15*.


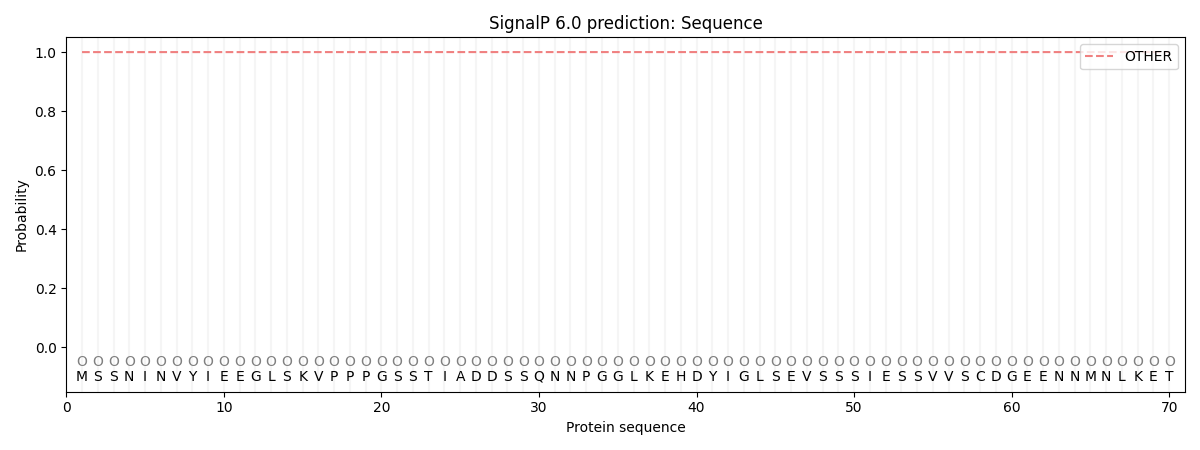


A


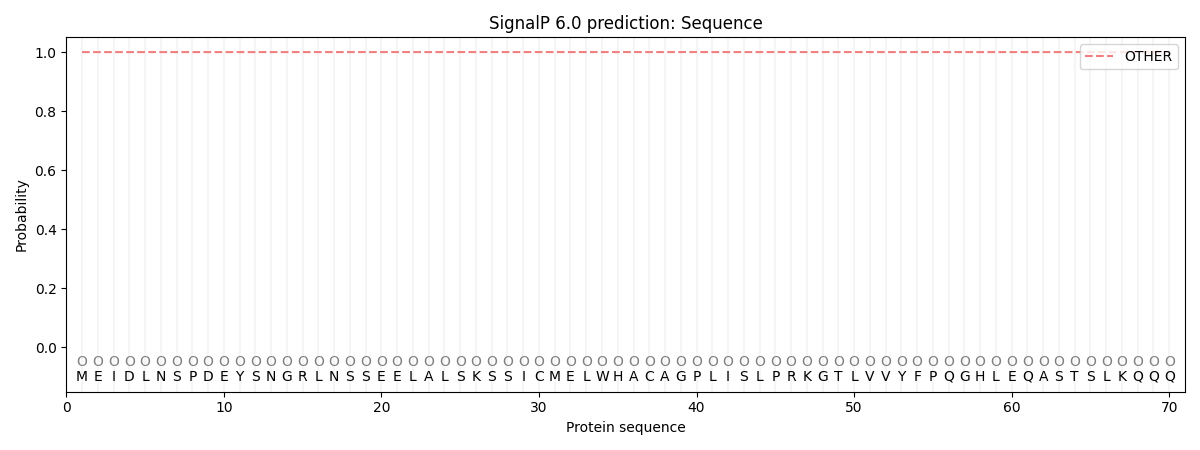


B

Fig. 4 signALP-4.1 results. A: *PmaIAA27*. B：*PmaARF15*.


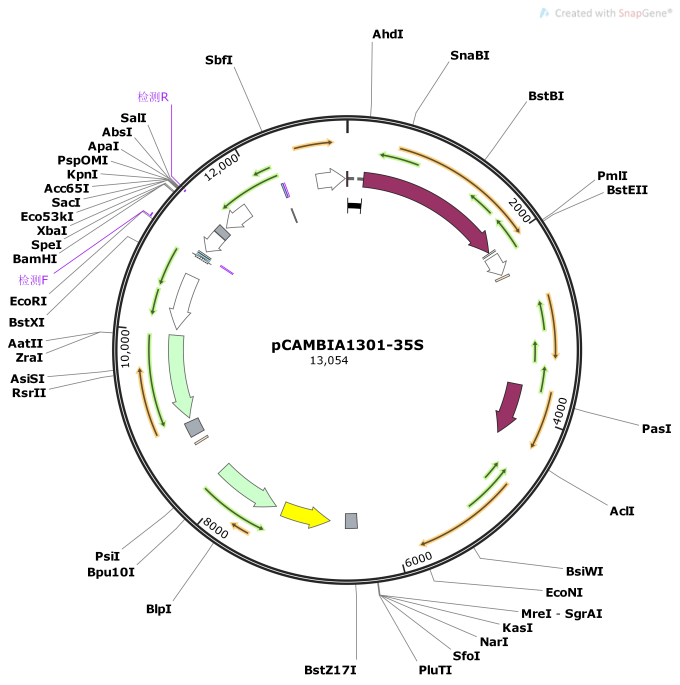


Fig. 5 Map of overexpression vector pCAMBIA1301


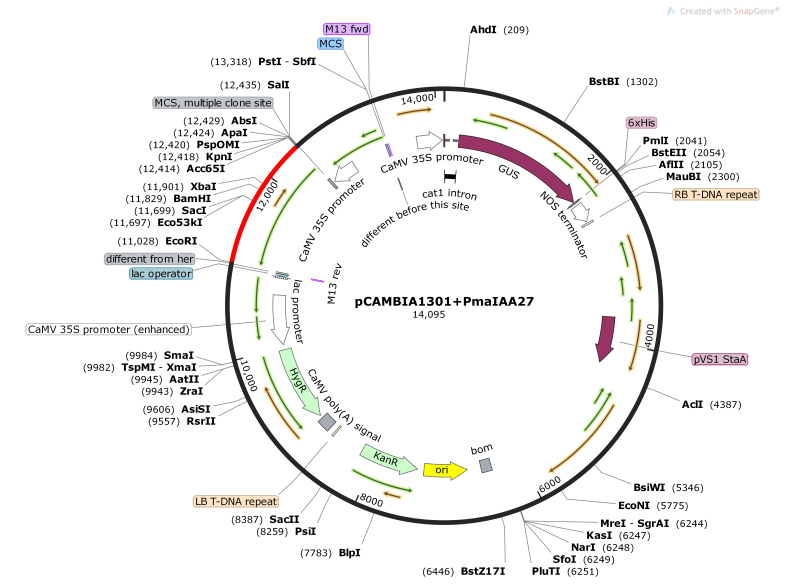


Fig. 6 Map of overexpression vector pCAMBIA1301+*PmaIAA27*


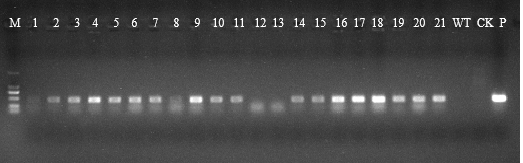


Fig. 7 17 positive strains (2-7,9-11,14-21 were positive).

Note: M is DL2000, WT for the negative control (wild type), CK for blank control (water),

P is a positive control (*Rhizobium radiobacter* solution)

Table 1 Experimental group and control group of yeast two-hybrid test

| Component | Experimental group | Positive control | Negative control |
| --- | --- | --- | --- |
| 100 ng pGBKT7-bait plasmid | + | - | - |
| 100 ng pGADT7-prey plasmid | + | - | - |
| 100 ng pGBKT7-53 plasmid | - | + | - |
| 100 ng pGBKT7-lam plasmid | - | - | + |
| 100 ng pGADT7-T plasmid | - | + | + |
| Y2H Gold yeast receptor cell | + | + | + |
| DDO tablet | + | + | + |
| TDO/3, AT tablet | + | + | + |
| QDO tablet | + | + | + |

Table 2 Gene-specific primers used in RT‒qPCR assays

| Gene | Primer sequences | |
| --- | --- | --- |
| *UBC* | F:AGGATCATCAGGATTTGGGT | R:GATTTATTTCATTGGCAGGC |
| *PmaARF15* | F:AGGAGACAAAGAGGAAGAGGA | R:CAAGGGAGGAAAGCAATCT |
| *PmaIAA27* | F: CCTTATTTGTCTTGGGCATC | R: AAGTCCCTCTCCCTTCAGTC |
